# Wnt signaling in breast cancer: biological mechanisms, challenges and opportunities

**DOI:** 10.1186/s12943-020-01276-5

**Published:** 2020-11-24

**Authors:** Xiufang Xu, Miaofeng Zhang, Faying Xu, Shaojie Jiang

**Affiliations:** 1 School of Medical Imaging, Hangzhou Medical College, Hangzhou, 310053 Zhejiang China; 2 Department of Orthopedic Surgery, Second Affiliated Hospital, School of Medicine, Zhejiang University, Hangzhou, 310009 Zhejiang China

**Keywords:** Canonical/noncanonical Wnt signaling, Breast cancer, Epithelial-mesenchymal transition, Drug resistance, Phenotype shaping, Immune microenvironment, Tumoral heterogeneity

## Abstract

Wnt signaling is a highly conserved signaling pathway that plays a critical role in controlling embryonic and organ development, as well as cancer progression. Genome-wide sequencing and gene expression profile analyses have demonstrated that Wnt signaling is involved mainly in the processes of breast cancer proliferation and metastasis. The most recent studies have indicated that Wnt signaling is also crucial in breast cancer immune microenvironment regulation, stemness maintenance, therapeutic resistance, phenotype shaping, etc. Wnt/β-Catenin, Wnt–planar cell polarity (PCP), and Wnt–Ca^2+^ signaling are three well-established Wnt signaling pathways that share overlapping components and play different roles in breast cancer progression. In this review, we summarize the main findings concerning the relationship between Wnt signaling and breast cancer and provide an overview of existing mechanisms, challenges, and potential opportunities for advancing the therapy and diagnosis of breast cancer.

## Background

Breast cancer was the most commonly diagnosed cancer (24.2% of the total cancer cases) and the leading cause of cancer-related death (15% of the total cancer deaths) among females worldwide in 2018 [1]. Metastatic disease accounts for more than 90% of breast cancer-related deaths [2]. Increasing evidence suggests that the genetic mutation-driven activation of Wnt signaling is the key factor in breast cancer metastasis [3].

Wnt signaling is an evolutionarily conserved pathway in metazoan animals [4]. The name ‘Wnt’ is a fusion of the name of the vertebrate homolog *Integrated* (*Int-1*) [5, 6] and the name of the *Drosophila* segment polarity gene *Wingless* [7, 8]. It has been almost four decades since the discovery of the *Int-1* proto-oncogene, now known as *Wnt-1*, which was identified as an integration site for mouse mammary tumor virus (MMTV) [5]. Breast cancer, on the other hand, is the first cancer to be associated with Wnt signaling.

In recent decades, a growing number of studies have demonstrated that Wnt signaling involves the proliferation [9], metastasis [3, 10, 11], immune microenvironment regulation [3, 12], stemness maintenance [13], therapeutic resistance [14], and phenotype shaping [15, 16] of breast cancer. Various Wnt signaling inhibitors that act on different targets have been developed, and many of them exhibit potent anticancer potential [17]. However, no Wnt inhibitors have been approved for breast cancer treatment to date.

This review describes the three well-established Wnt signaling pathways, summarizes the main findings between Wnt signaling and breast cancer based on biological mechanisms, elaborates the challenges in drugging Wnt signaling, and provides potential solutions for both basic research and the clinical treatment of breast cancer.

### An overview of the Wnt signaling pathway

There are 19 Wnt genes in the human genome, all of which encode secreted lipoglycoproteins that have fundamental roles in controlling cell specification, cell-cell interactions, stem cell self-renewal, and tissue patterning during embryonic development [18, 19]. Wnt proteins (Wnts) couple to various receptors and thereby activate different downstream pathways [20]. Canonical Wnt signaling is a β-Catenin-dependent and T cell factor (TCF)/lymphoid enhancer factor (LEF)-involved pathway that is responsible mainly for breast cancer cell proliferation and ‘stemness’ maintenance [21]. Increasing evidence indicates that Wnt–PCP and Wnt–Ca^2+^ signaling, the two well-established β-Catenin-independent noncanonical Wnt pathways, are responsible for breast cancer cell metastasis [22, 23]. The processes of immune microenvironment regulation, therapeutic resistance, and phenotype shaping of breast cancer seem complicated and are always mediated by cooperation and crosstalk between canonical and noncanonical Wnt pathways.

### Canonical Wnt signaling pathway

Canonical Wnt signaling (also known as Wnt/β-Catenin signaling) is the best-characterized pathway and is generally triggered by Wnt1, Wnt2, Wnt3, Wnt3a, Wnt8b, Wnt10a, Wnt10b, and so on (Table 1) [18, 19, 79]. In the endoplasmic reticulum (ER), the conserved cysteine of Wnts is palmitoylated by Porcupine to a lipid-bound form that is also an active form [80]. The ER-to-Golgi trafficking of Wnts is mediated by the p24 protein family (such as TMED2/CHOp24, TMED4/éclair, and TMED5/opossum) [81–83]. Then, lipid-modified Wnts are transported by GPR177 (also known as Wntless/Evenness/Interrupted/Sprinter) [84–87] in an endosome-dependent manner [88–90] and secreted into the extracellular matrix using exosomes as potent carriers [10, 91–93]. Notum is a deacetylase that acts as a lipid eraser of Wnts and can inactivate Wnts [94, 95].

**Table 1:** Wnt ligands and related factors in breast cancer

Ligand	Signaling pathway	Alterations in breast cancer	Ref.
Wnt1	Canonical	Activated by MMTV integration in breast cancer; Activated by *TP53* loss in breast cancer; Highly expressed in breast cancer	[3, 5, 24–26]
Wnt2	Canonical	Expressed at a high level in breast cancer	[27–32]
Wnt2b	Canonical	–	[33, 34]
Wnt3	Canonical	Overexpressed in trastuzumab-insensitive breast cancer cells; Activated by TGFβ in breast cancer cells	[35–37]
Wnt3a	Canonical	Amplified in breast cancer	[16]
Wnt4	Noncanonical	Driven by estrogen and progesterone in breast cancer	[25, 27, 38, 39]
Wnt5a	Canonical/noncanonical	Highly expressed in BLBC	[15, 16, 22, 40]
Wnt5b	Canonical/noncanonical	Highly expressed in BLBC	[16, 22, 41–43]
Wnt6	Canonical	Activated by *TP53* loss in breast cancer	[3]
Wnt7a	Canonical/noncanonical	Activated by *TP53* loss in breast cancer; Secreted exclusively by aggressive breast cancer cells	[3, 44, 45]
Wnt7b	Canonical/noncanonical	Activated by TGFβ in breast cancer cells	[27, 45, 46]
Wnt8a	Noncanonical	–	[47]
Wnt8b	Canonical	–	[48]
Wnt9a	Canonical	Amplified in breast cancer	[16, 49]
Wnt9b	Canonical/noncanonical	–	[50–52]
Wnt10a	Canonical	Expressed in mouse ALDH-negative breast cancer cells in a time-dependent manner	[53]
Wnt10b	Canonical	Highly expressed in TNBC	[9, 54–56]
Wnt11	Canonical/noncanonical	Induced by ERα and β-Catenin	[57, 58]
Wnt16	Canonical/noncanonical	–	[59–62]
Porcupine	Canonical/noncanonical	–	[63]
p24 proteins	Canonical/noncanonical	TMED2 is increased in breast cancer	[64]
GPR177	Canonical/noncanonical	Markedly increased in breast cancer	[65]
Notum	Canonical/noncanonical	–	–
Norrin	Canonical/noncanonical	Significantly decreased in breast cancer	[66]
R-spondin	Canonical/noncanonical	R-spondin-1 is secreted by differentiated mammary luminal cells	[67, 68]
Cerberus	Canonical/noncanonical	–	–
sFRPs	Canonical/noncanonical	sFRP1, sFRP2, and sFRP5 are aberrantly methylated or epigenetically suppressed in breast cancer	[69–74]
WIF	Canonical/noncanonical	WIF-1 is epigenetically silenced or lost in breast cancer	[75, 76]
SOST	Canonical	Induced expression by Runx2/CBFβ in metastatic breast cancer cells	[77]
Dkks	Canonical/noncanonical	Dkk1 is epigenetically inactivated in breast cancer	[69]
IGFBP4	Canonical	Protease-resistant IGFBP4 is expressed in murine breast cancer	[78]

Frizzleds (Fzds) are 7-transmembrane (7-TM) proteins that act as the primary receptors for Wnts [96–98], while low-density lipoprotein receptor-related proteins (LRPs) are single-pass transmembrane proteins that act as coreceptors for Fzds [99–101]. Wnt signaling is inhibited by endogenous inhibitors, such as Wnt inhibitory factor 1 (WIF-1) [102], Cerberus [103], and secreted Fzd-related proteins (sFRPs) [104] that interact with Wnts directly, Wise/SOST [105–107] and dickkopf proteins (Dkks) [108, 109] that bind to LRPs and block Fzds–LRP heterodimer formation, and insulin-like growth factor-binding protein 4 (IGFBP4) physically interacts with Fzd8 and LRP6 and inhibits Wnt3a binding [110]. Of note, sFRPs can also interact with Fzds and inhibit Wnt signaling [111, 112]. Additionally, Rnf43 and Znrf3 are two single-pass transmembrane E3 ligases that specifically mediate the multiubiquitination of Fzds [113, 114].

Wnt signaling is maintained in an off state in the absence of extracellular Wnts. β-Catenin is the core component of canonical Wnt signaling and binds to the cytoplasmic tail of E-cadherin for cell-cell adhesion [115–118]. In the cytoplasm, β-Catenin is hijacked by the ‘destruction complex’, which comprises adenomatous polyposis coli (APC) [119, 120], Axin [121–124], glycogen synthase kinase 3β (GSK-3β) [125, 126], casein kinase 1α (CK1α) [127, 128], protein phosphatase 2A (PP2A) [129], and Wilms tumor gene on X chromosome (WTX) [130], thereby being ubiquitinated by the Skp1, Cullin1 and F-box protein β-TrCP (SCF^β-TrCP^) ubiquitin ligase and degraded [131, 132]. β-Catenin is first phosphorylated by CK1α at Ser45, followed by GSK-3β phosphorylation at the Thr41, Ser37, and Ser33 residues [90]. The phosphorylation of Ser33 and Ser37 creates the recognition site for β-TrCP [127] for subsequent degradation. Tankyrase 1/2 (TNKS1/2) destabilizes Axin, making it an attractive target for Wnt signaling regulation [133]. In addition, Siah-1 interacts with APC and promotes the degradation of β-Catenin independent of GSK-3β-mediated phosphorylation and β-TrCP-mediated ubiquitin [134].

In the nucleus, TCF [135, 136] and C-terminal binding protein (CTBP) [137] interact with Transducin-like enhancer/Groucho (TLE/GRG), while histone deacetylases (HDACs) interact with TCF and LEF1 [138, 139]. These proteins form a repressor complex that represses the expression of Wnt target genes [140]. In addition, β-Catenin is inhibited from binding to TCF/LEF by inhibitors of β-Catenin and TCF (ICAT) [141] and Chibby (CBY) [142].

The canonical Wnt signaling cascade is initiated from the binding of lipid-modified Wnts to the receptor complex. Norrin binds to Fzd4 and activates the canonical Wnt pathway, although it is structurally unrelated to Wnts [143–145]. On the other hand, R-spondin binds to leucine-rich repeat-containing G protein-coupled receptor 5 (LGR5) and induces the membrane clearance of Rnf43/Znrf3, which removes the ubiquitylation of Fzd4 [113, 114]. LRP6 is phosphorylated by GSK-3 and CK1 [146, 147], which recruits the scaffold protein Axin [148], while Fzds recruit Dishevelled (Dvl) [149] to the plasma membrane, thereby disrupting the destruction complex [150].

**Fig. 1:**
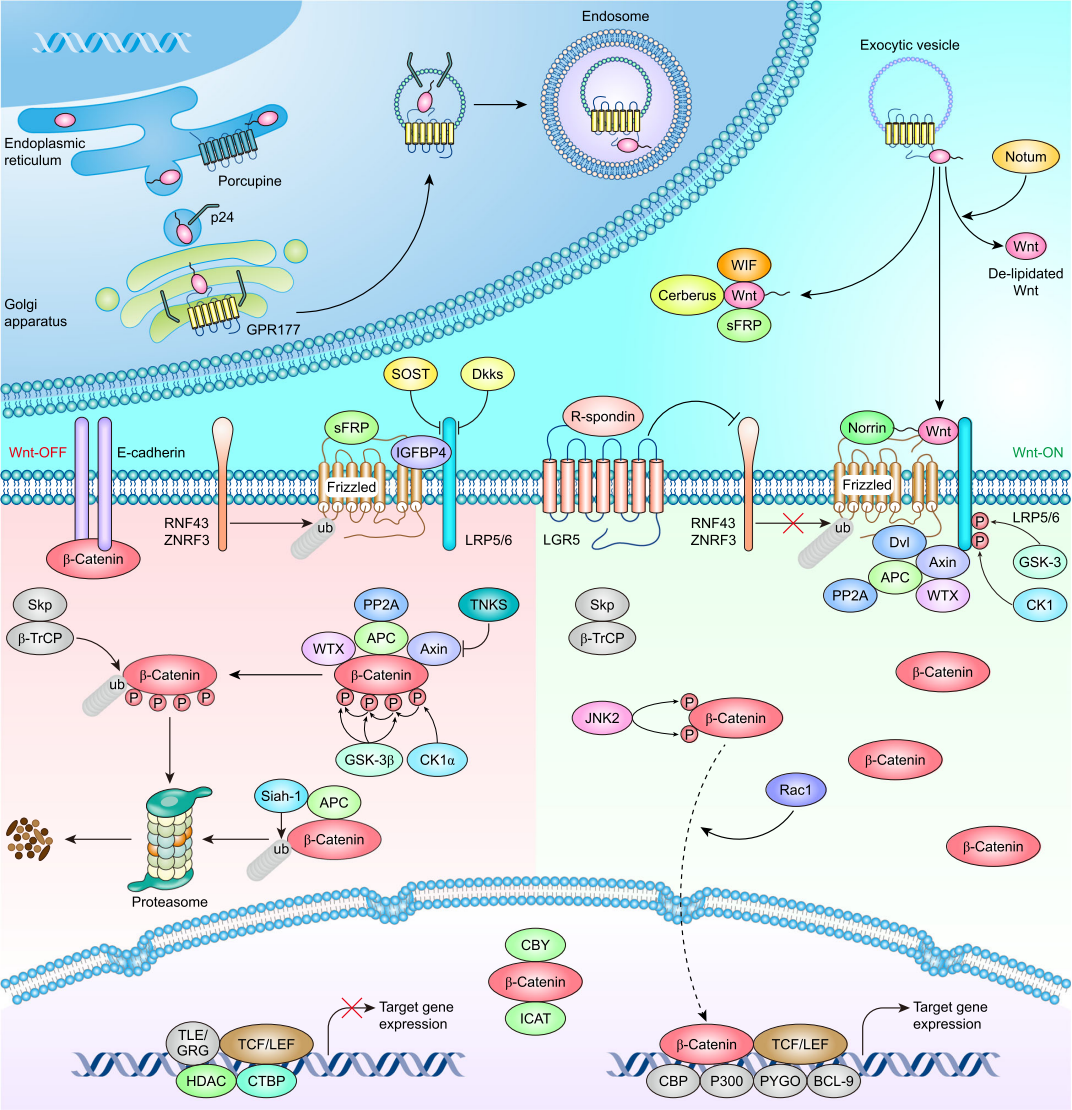
Canonical Wnt signaling pathway in mammals

β-Catenin is phosphorylated at Ser191 and Ser605 by Jun N-terminal kinase 2 (JNK2), which facilitates its nuclear localization mediated by Rac1 [151]. In the nucleus, β-Catenin serves as a scaffold for the LEF [152, 153] and TCF [154–156] families, recruiting coactivators such as CREB-binding protein (CBP)/p300 [157], Pygopus (PYGO) and B cell lymphoma 9 (BCL9) [158, 159] and leading to the transcription of a large set of target genes (Fig. 1).

### Wnt–PCP signaling pathway

Wnt–PCP signaling does not involve β-Catenin, LRP, or TCF molecules and is generally triggered by Wnt4, Wnt5a, Wnt5b, Wnt7b, and Wnt11 [160–162] (Table 1). These Wnts can also be inhibited by directly binding to endogenous inhibitors, including sFRPs, WIF, and Cerberus, and sFRPs may also inhibit Wnt–PCP signaling by binding to Fzds [163].

**Fig. 2:**
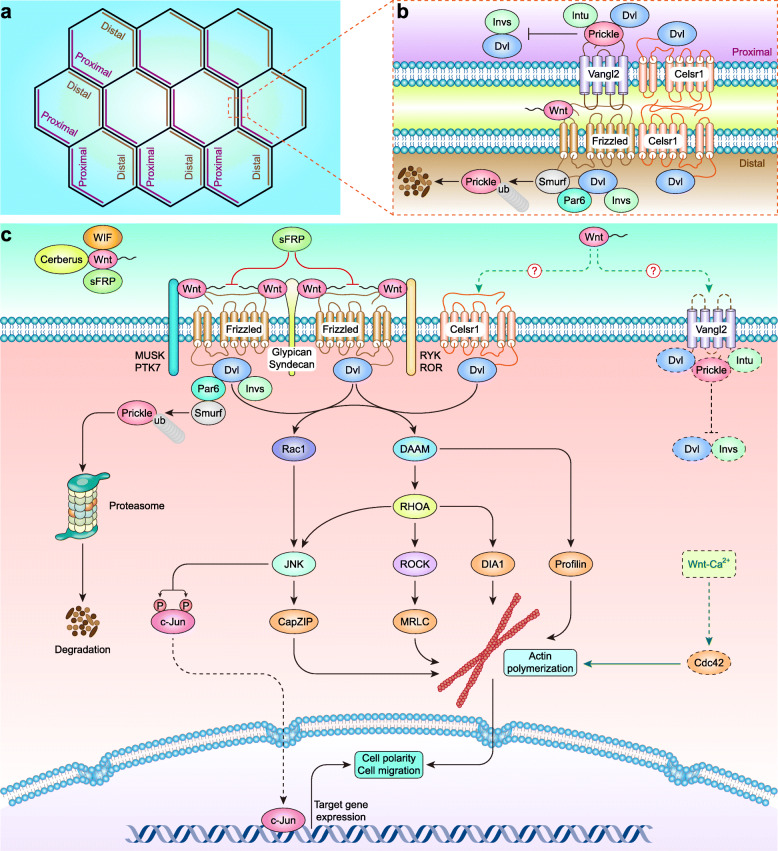
Wnt–PCP signaling pathway in mammals. **a** Planar cell polarity of the asymmetric transmembrane complexes. **b** Asymmetric PCP signaling components form transmembrane complexes. **c** Wnt–PCP signaling pathway in mammals

The complementary and mutually exclusive distribution of transmembrane complexes is the key feature of planar polarization, which results in the asymmetric enrichment of proximal and distal transmembrane complexes in cells (Fig. 2a). The proximal transmembrane complex is composed of Vang-like 2 (Vangl2), cadherin EGF LAG seven-pass G-type receptor 1 (Celsr1), Prickle, inturned (Intu) and Dvl [164], while the distal transmembrane complex is composed of Fzds, Celsr1, Inversin (Invs) and Dvl. On the proximal side, Vangl2 recruits Prickle, which competes with Invs for Dvl binding and therefore disrupts the localization of Invs towards the proximal side. Intriguingly, noncanonical Wnt binding to Fzds leads to Dvl phosphorylation and distal side localization of the Invs [165] and Dvl–Par6 complex, and Smurf is recruited by phosphorylated Dvl to Par6, thereby ubiquitinating and degrading Par6-bound Prickle on the distal side and antagonizing the inhibitory action of Prickle on Wnt–PCP signaling [166] (Fig. 2b). This asymmetric cell patterning, in turn, directs the orientation of subcellular structures and cell behaviors through the regulation of cytoskeletal elements and cellular adhesions [161].

Fzds [167–170], Celsr1 [171–173], and Vangl2 [174–179] are core receptors in Wnt–PCP signaling. Fzds are still the primary receptors for Wnts, while receptor-like tyrosine kinase (RYK) [180], muscle-skeletal receptor Tyr kinase (MUSK) [181–183], protein tyrosine kinase 7 (PTK7) [184], receptor Tyr kinase-like orphan receptor 1/2 (ROR1/2) [162], Syndecan [185, 186] and Glypican [187] act as coreceptors for Fzds. However, the ligand-receptor binding interaction between Wnts and Celsr1 [188] or Vangl2 [189] has not been clarified to date.

The formation of the Fzds–Celsr1–Invs–Dvl complex and the interaction between Dvl and Dvl-associated activator of morphogenesis (DAAM) activate the small GTPases Rac1 [190] and Ras homologue gene-family member A (RHOA) [191, 192]. Rac1 activates JNK [193], which further phosphorylates c-Jun at Ser63 and Ser73, thereby activating c-Jun [194]. RHOA subsequently activates diaphanous 1 (DIA1) and RHO-associated coiled-coil-containing protein kinase (ROCK) [195]. Then, JNK activates CapZ-interacting protein (CapZIP) [196], ROCK activates mitogen-activated protein kinase (MRLC) [197], and DAAM activates Profilin [198]. These PCP effectors lead to the development of lateral asymmetry in epithelial sheets and other structures [199], as well as cell polarity and migration by remodeling the cytoskeleton [200] (Fig. 2c).

### Wnt–Ca^2+^ signaling pathway

**Fig. 3:**
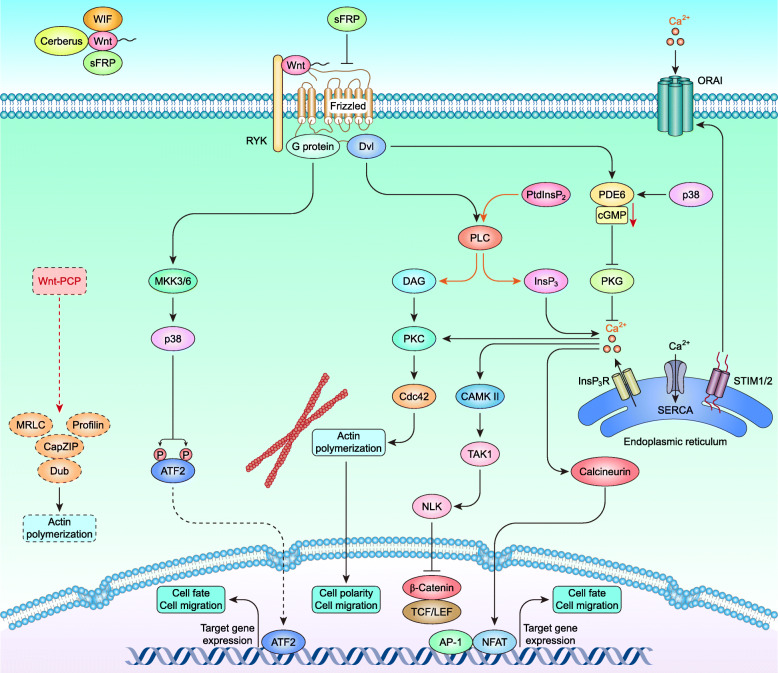
Wnt–Ca^2+^ signaling pathway in mammals

Wnt–Ca^2+^ signaling is a less focused noncanonical Wnt pathway but plays a central role in cell fate during early embryogenesis [201], cancer progression [202–204], interneural communication [205], the inflammatory response [206], and so on. Wnt–Ca^2+^ signaling is initiated mostly by Wnt5a and Fzd2, and pertussis toxin-sensitive heterotrimeric G protein subunits [207] are required for the activation of phospholipase C (PLC). Activated PLC cleaves phosphatidylinositol-4,5-bisphosphate (PtdInsP_2_), a membrane-bound inositol lipid, into diacylglycerol (DAG) and inositol-1,4,5-trisphosphate (InsP_3_). DAG, together with Ca^2+^, activates protein kinase C (PKC), which further stimulates cell-division cycle 42 (Cdc42) and promotes actin polymerization. On the other hand, InsP_3_ binds to inositol-1,4,5-trisphosphate receptors (InsP_3_Rs) [208] on the ER membrane, opening calcium channels for Ca^2+^ release and increasing cytoplasmic Ca^2+^ levels [209]. The decrease in Ca^2+^ levels within the ER lumen is sensed by stromal interaction molecule 1/2 (STIM1/2), which gains an extended conformation to trap and activate ORAI proteins at the plasma membrane and induce store-operated Ca^2+^ entry (SOCE) [210, 211]. In addition, sarcoplasmic/ER Ca^2+^ ATPases (SERCAs) act as Ca^2+^ ion pumps that pump Ca^2+^ from the cytosol to the ER. An increased Ca^2+^ concentration activates the phosphatase calcineurin and several calcium-dependent kinases, including PKC and calcium calmodulin mediated kinase II (CAMKII). The increased activity of calcineurin, in turn, activates the nuclear factor of activated T cells (NFAT) [212]. In contrast, CAMKII stimulation activates TGFβ-activated protein kinase 1 (TAK1), which subsequently activates nemo-like kinase (NLK), resulting in the phosphorylation of TCF and the inhibition of β-Catenin/TCF signaling [213, 214]. The Wnt–Fzd–Dvl complex also activates cyclic guanosine monophosphate (cGMP)-specific phosphodiesterase 6 (PDE6), thereby depleting cellular cGMP and inactivating protein kinase G (PKG), which in turn increases the cellular concentration of Ca^2+^. The G protein-induced activation of p38 via mitogen-activated protein kinase 3/6 (MKK3/6) is required for the activation of PDE6. Moreover, the p38-induced phosphorylation of activating transcription factor 2 (ATF2) on Thr69 and Thr71 is important for its transcription [215, 216] (Fig. 3).

### Wnt signaling alterations in breast cancer

Numerous studies have shown that the constitutive components of Wnt signaling are altered in breast cancer cells. These alterations include mutations, amplifications, deletions, and methylations that occur at the DNA level, posttranscriptional modifications that occur at the mRNA level, and posttranslational modifications that occur at the protein level. These alterations also include changes in subcellular localization, especially for β-Catenin. Mutation of *CTNNB1*, which encodes β-Catenin, is rare in breast cancer [217]. However, the activation of Wnt signaling is nonetheless thought to play an essential role in breast tumorigenesis [69]. This is mainly due to the epigenetic activation of Wnts and the inactivation of Wnt inhibitors (Table 1). Nonetheless, it has been reported that Wnt5a is lost in breast cancer [75, 218–220]. Foxy-5, a Wnt5a mimicking hexapeptide, impairs the migration and invasion of breast cancer without affecting apoptosis or proliferation by reconstituting Wnt5a signaling [221] and has entered phase I clinical trials (NCT02020291 and NCT02655952) for metastatic breast, colorectal and prostate cancer treatment and a phase II clinical trial (NCT03883802) for Wnt5a-low colon cancer neoadjuvant therapy. In addition, most canonical and noncanonical Wnt receptors are elevated in breast cancer, especially in triple-negative breast cancer (TNBC) and basal-like breast cancer (BLBC). E-cadherin, as an interacting protein of β-Catenin, is frequently mutated or silenced in BLBC and TNBC, which leads to the release of β-Catenin from the cytomembrane into the cytoplasm [222–224] (Table 2).

**Table 2:** Wnt receptors and coreceptors in breast cancer

Receptor	Signaling pathway	Alterations in breast cancer	Ref.
Fzd1	Canonical	Upregulated in breast cancer	[225, 226]
Fzd2	Noncanonical	Elevated in metastatic breast cancer	[22, 225]
Fzd3	–	–	–
Fzd4	–	–	–
Fzd5	–	–	–
Fzd6	Noncanonical	Genomically amplified in TNBC	[227]
Fzd7	Canonical	Elevated in BLBC/TNBC	[228–230]
Fzd8	–	–	–
Fzd9	–	Relatively hypermethylated in breast cancer	[231]
Fzd10	–	–	–
LRP5	Canonical	–	–
LRP6	Canonical	Markedly upregulated in breast cancer; Overexpressed in TNBC	[229, 232]
RNF43	Canonical	–	–
ZNRF3	Canonical	–	–
E-cadherin	Canonical	Mutated or silenced in BLBC/TNBC	[222–224]
LGR5	Canonical	Overexpressed in breast cancer	[233, 234]
Celsr1	Noncanonical	Highly expressed in luminal breast cancer	[235]
Vangl2	Noncanonical	Highly expressed in BLBC	[189]
ROR1	Noncanonical	Highly expressed in TNBC, BLBC, and aggressive/metastasis-prone breast cancer	[236–240]
ROR2	Canonical/noncanonical	Highly expressed in breast cancer	[241]
RYK	Noncanonical	Reduced in primary breast cancer	[220]
PTK7	Noncanonical	Elevated in TNBC and BLBC	[242–244]
MUSK	Noncanonical	–	–
Syndecan	Noncanonical	Syndecan-1 is overexpressed in breast cancer; Induced expression in stromal fibroblasts of breast cancer	[245–248]
Glypican	Noncanonical	Glypican-3 is silenced in human breast cancer	[249]
ORAI	Noncanonical	ORAI1 is elevated in BLBC; ORAI3 is elevated in breast cancer	[250–253]

Cytoplasmic β-Catenin should be carefully controlled by the destruction complex in the cytoplasm. However, the destruction complex components are frequently mutated, deleted, hypermethylated, or reduced in breast cancer, which increases the stability of cytoplasmic β-Catenin and the probability of β-Catenin entering the nucleus. Most coactivators are highly expressed in breast cancer, as expected. However, it is interesting that some corepressors (e.g., TLE/GRG and CTBP) are elevated in breast cancer [254, 255] (Table 3). These studies suggest that the activation of canonical Wnt signaling in breast cancer is induced mainly by epigenetic alterations in the constitutive components rather than the mutation of β-Catenin or APC. Noncanonical Wnt signaling is preferentially activated in TNBC/BLBC and is induced mainly by the epigenetic activation of noncanonical Wnts and their receptors (Tables 1 & 2). Cytoplasmic components of noncanonical Wnt signaling are commonly involved in various signaling pathways and are challenging to define as exclusive components of noncanonical Wnt signaling (Figs. 2 & 3).

**Table 3:** β-Catenin and its related factors in breast cancer

Protein	Function	Alterations in breast cancer	Ref.
β-Catenin	Key mediator	Increased nuclear accumulation in TNBC and BLBC; Activation is enriched in BLBC	[256, 257]
APC	Destruction complex	Mutated, deleted, hypermethylated or reduced in breast cancer	[258–262]
PP2A		Reduced activity in breast cancer	[263]
WTX		Reduced in breast cancer	[264]
Axin		Mutated in breast cancer	[265]
GSK-3β		Reduced in BLBC cells	[16]
CK1α		Reduced in BLBC cells	[16]
Siah-1		–	–
TNKS	Destabilizer of Axin	Overexpressed in breast cancer	[266]
JNK2	Transcriptional cofactor	–	–
Rac1		Mutated and overexpressed in breast cancer	[267]
TLE/GRG	Corepressor	TLE1 is selectively upregulated in invasive breast cancer	[254]
HDAC		–	–
CTBP		Elevated in TNBC and BLBC	[255]
CBY		–	–
ICAT		–	–
TCF	Coactivator	TCF1/7 is overexpressed in BLBC cells	[16]
LEF		LEF1 is highly expressed in Her-2-negative breast cancer	[268]
CBP		–	–
P300		Highly expressed in breast cancer	[269]
PYGO		PYGO2 is highly expressed in breast cancer cells	[270]
BCL-9		Significantly amplified in BLBC	[271]

### Wnt signaling in breast cancer classification

**Fig. 4:**
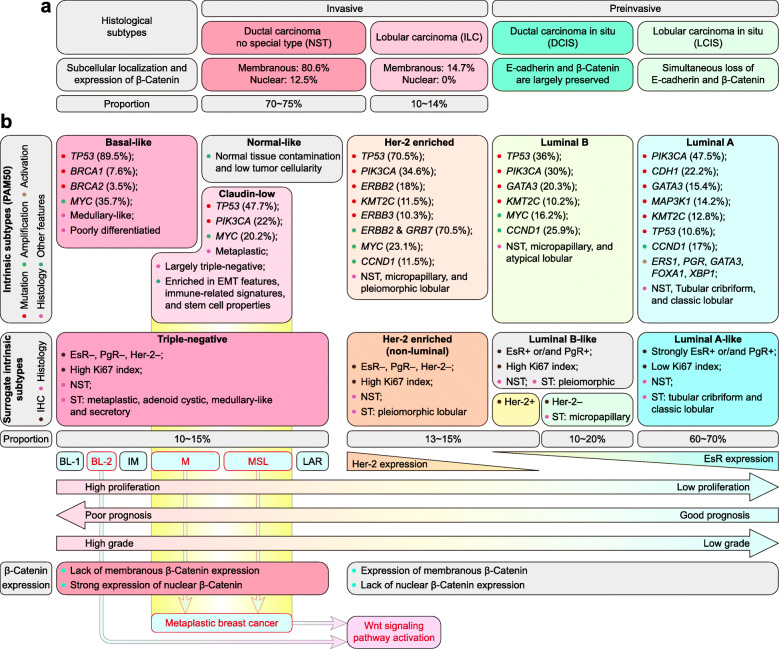
Wnt signaling in breast cancer classification. **a** Wnt signaling in the histological classification of breast cancer. **b** Wnt signaling in the molecular classification of breast cancer. (adapted from [272], additional data are based on an open-source database: www.cbioportal.org)

Invasive ductal carcinoma no-special-type (IDC-NST) and invasive lobular carcinoma (ILC) are the most common histological subtypes of breast carcinoma, accounting for 70 ~ 75% and 10 ~ 14%, respectively [272]. β-Catenin expression is significantly correlated with histological type. The majority of IDCs display a regular pattern of β-Catenin expression, with membranous expression (80.6%) and nuclear expression (12.5%), whereas ILCs lack membranous expression (14.7%) and nuclear expression (0%) [256]. Additionally, E-cadherin and β-Catenin expression is largely preserved in ductal carcinoma in situ (DCIS) [273]. However, lobular carcinoma in situ (LCIS) shows the simultaneous loss of E-cadherin and β-Catenin expression [274]. This may explain why IDCs have a worse prognosis than ILCs [275] (Fig. 4a).

Furthermore, breast cancer can be classified into five intrinsic subtypes: luminal A, luminal B, Her-2 enriched, normal-like, and BLBC [276–278]. However, the normal-like subtype is controversial, potentially due to normal tissue contamination and low tumor cellularity [279]. Claudin-low was initially considered a breast cancer subtype [280, 281] and later redefined as a breast cancer phenotype [282].

Surrogate intrinsic subtypes are based on the immunohistochemistry of estrogen receptor (EsR), progesterone receptor (PgR), human epidermal growth factor receptor 2 (Her-2), and Ki67 and consist of luminal A-like, luminal B-like, Her-2 enriched, and TNBC [283]. The majority of BLBCs and claudin-low intrinsic subtypes are TNBCs [272, 284]. TNBCs can be further divided into basal-like 1 (BL-1), basal-like 2 (BL-2), immunomodulatory (IM), luminal androgen receptor (LAR), mesenchymal (M), and mesenchymal stem-like (MSL) [285].

The claudin-low subtype is composed mostly of the M and MSL subtypes of TNBC [286], which, together with the BL-2 subtype of TNBC, are linked to Wnt signaling activation [285]. Intriguingly, the majority of claudin-low cancers are metaplastic breast cancers [281]. This is consistent with the previous description that Wnt signaling activation is enriched in metaplastic breast cancers [287], BLBCs [257], and TNBCs [256]. Specifically, Wnt signaling activation in metaplastic breast cancers is caused mainly by genetic changes, such as *CTNNB1* and *APC* mutations [287], whereas Wnt signaling activation in BLBCs and TNBCs is associated mainly with the strong expression of nuclear β-Catenin [256, 257]. This may be the main reason for the worst prognosis of TNBCs among all subtypes. Notably, both basal and luminal tumor cells are found in MMTV-Wnt1 mammary tumors, implying that they are derived from a bipotent malignant progenitor cell [24, 288]. Based on these studies, Wnt signaling is the critical pathway for phenotype shaping in both histological subtypes and molecular subtypes of breast cancer (Fig. 4 b).

### Wnt signaling in the breast cancer immune microenvironment

**Fig. 5:**
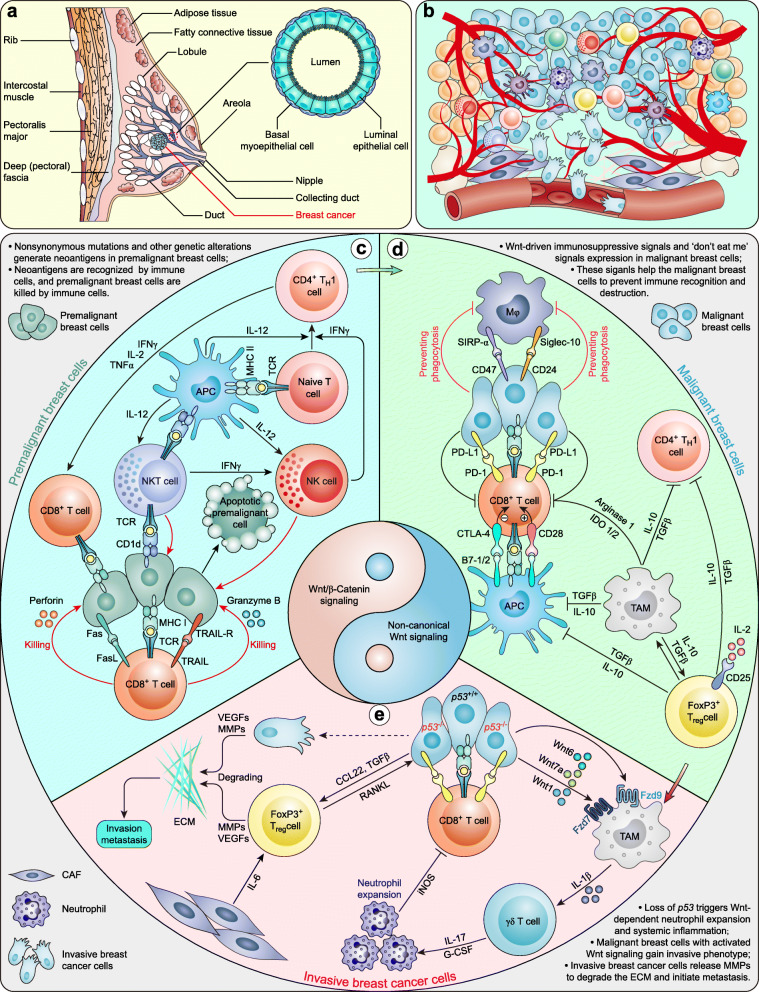
Wnt signaling in the immune microenvironment of breast cancer. **a** Schematic representation of the human mammary gland, breast cancer, and an enlarged cross-section of the duct (adapted from [272]). **b** Tumor microenvironment of breast cancer. **c** Wnt signaling in the immune microenvironment of breast cancer

All breast cancers arise in the terminal duct lobular units (the functional unit of the breast) of the collecting duct [272] (Fig. 5 a). Breast cancer cells commonly reside in a complicated tumor microenvironment that is composed mainly of genetically abnormal cells surrounded by blood vessels, fibroblasts, immune cells, stem cells and the extracellular matrix (ECM), and dynamic crosstalk among these various components ultimately determines the fate of breast cancer [289] (Fig. 5 b).

The reciprocal crosstalk between breast cancer cells and immune cells is initiated by the neoantigens [272] that arise from nonsynonymous mutations and other genetic alterations [290]. These neoantigens are presented by antigen-presenting cells (APCs) on major histocompatibility complex class I (MHC I) or MHC II molecules [272], resulting in the activation of CD8^+^ (cytotoxic) [291] and CD4^+^ (helper) T cells [292]. Activated CD8^+^ T cells directly induce premalignant breast cell lysis by releasing cytolytic perforin and granzyme B [293] and promote the apoptosis of premalignant breast cells by expressing Fas ligand (FasL) and TNF-related apoptosis-inducing ligand (TRAIL) on their cell surface [272, 294]. This may explain why high breast tumor-infiltrating CD8^+^ T cell counts are associated with improved clinical outcomes [295]. CD4^+^ T_H_1 cells arise from naive T cells that are activated by interleukin (IL)-12 (provided by dendritic cells and macrophages) and interferon (IFN)-γ (provided by natural killer (NK) cells) [296] and amplify the anticancer effect of CD8^+^ T cells by secreting IFN-γ, IL-2, and tumor necrosis factor (TNF)-α. NK T cells recognize MHC I-like molecule CD1d on dendritic cells and are further activated by IL-12 that is expressed by dendritic cells. Activated NK T cells also recognize CD1d expressed by breast cancer cells [297] and recruit NK cells by releasing IFN-γ, which kills breast cancer cells directly (Fig. 5 c).

However, Wnt/β-catenin signaling activation suppresses the antitumor immune response [298]. Malignant breast cells with activated Wnt signaling develop several strategies to avoid immune recognition and destruction. They express CD24 [299] and CD47 [300–302] as ‘don’t eat me’ signals to prevent phagocytosis from macrophages by interacting with Siglec-10 and SIRP-α, respectively, expressed by macrophages. Remarkably, CD24 is a direct target of Wnt1 in breast cancer [303], while CD47 is an indirect target of Wnt signaling mediated by SNAI1 and ZEB1 in breast cancer [304]. In addition, CD47 and programmed death-ligand 1 (PD-L1) are controlled by *Myc*, a well-documented target of Wnt/β-catenin signaling [305]. In addition, TNBCs upregulate PD-L1 through Wnt signaling activity, thereby blocking CD8^+^ T cell activation [306]. Cytotoxic T lymphocyte antigen 4 (CTLA-4) is expressed at a low level in naive T cells but is rapidly induced after activation. Coincidentally, CTLA-4 is also a direct target of Wnt/β-catenin signaling [307]. Tumor-associated macrophages (TAMs) [308] and forkhead box protein P3 (FoxP3)^+^ T_reg_ cells [309–311] are commonly associated with a poor clinical outcome. TAMs directly inhibit T cell functions by expressing checkpoint ligands (such as PD-L1, PD-L2, B7–1/CD80, and B7–2/CD86) [312] and inhibit CD4^+^ T_H_1, T_H_2, and CD8^+^ T cells by secreting immunosuppressive cytokines (such as IL-10 and TGFβ) [313]. TAMs also inhibit cytotoxic T cells by releasing arginase 1 and indoleamine 2,3-dioxygenase (IDO). In addition, TAMs secrete Wnt7b, which mediates the angiogenic switch and metastasis in breast cancer [46]. FoxP3^+^ T_reg_ cells exert their immunosuppressive effect by consuming IL-2, secreting immunosuppressive cytokines (such as IL-10, IL-35, and TGFβ), converting ATP into adenosine, and secreting perforin and/or granzyme B, thereby limiting, inhibiting or destroying effector cells and attenuating the functions of APCs mediated by CTLA-4 [314]. Intriguingly, TAMs and FoxP3^+^ T_reg_ cells express IL-10 and TGFβ, which induces reciprocal activation. Based on these studies, atezolizumab, an anti-PD-L1 monoclonal antibody, has been approved by the Food and Drug Administration (FDA) for advanced or metastatic TNBC with PD-L1 expression [315]. T cell exhaustion mediated by Wnt signaling is a strategy that was developed for the immune escape of malignant breast cells (Fig. 5 d).

*TP53* is the most frequently altered gene in metastatic breast cancers [316]. The loss of *TP53* in breast cancer cells triggers the secretion of Wnt1, Wnt6, and Wnt7a. These Wnts bind to Fzd7 and Fzd9 on the surface of TAMs, stimulating TAMs to produce IL-1β [3]. IL-1β elicits IL-17 expression from γδ T cells, resulting in the systemic, granulocyte colony-stimulating factor (G-CSF)-dependent expansion and polarization of neutrophils. Phenotypically altered neutrophils produce inducible nitric oxide synthase (iNOS), which suppresses the activity of antitumor CD8^+^ T cells and thereby induces systemic inflammation and drives breast cancer metastasis [317]. FoxP3^+^ T_reg_ cells express receptor activator of nuclear factor-κB (RANK) ligand (RANKL), which stimulates the pulmonary metastasis of RANK^+^ breast cancer cells [318]. Cancer-associated fibroblasts (CAFs) promote tumor immunosuppression by releasing IL-6, which increases the number of FoxP3^+^ T_reg_ cells [319]. Malignant breast cells acquire invasive properties and become invasive breast cancer cells through an epithelial-mesenchymal transition (EMT)-dependent process that is mediated mostly by the Wnt signaling pathway [320–323] (see below). FoxP3^+^ T_reg_ cells and invasive breast cancer cells secrete matrix metalloproteinases (MMPs) and vascular endothelial growth factors (VEGFs) to degrade the ECM and promote angiogenesis, resulting in breast cancer metastasis (Fig. 5 e). Of note, MMPs and VEGFs are classic targets of Wnt signaling [324]. Collectively, these findings show that Wnt-driven systemic inflammation and the immunosuppressive niche provide an immune microenvironment for breast cancer metastasis.

### Wnt signaling in the EMT-dependent metastasis of breast cancer

The immune microenvironment is an external factor in breast cancer metastasis, while genetic alteration-driven cell transformation is the internal factor in breast cancer metastasis [325]. Increasing evidence suggests that EMT contributes to the primary cause of breast cancer metastasis, especially for BLBCs [326]. The term EMT refers to a complicated and highly regulated molecular and cellular process by which epithelial cells shed their differentiated characteristics and acquire mesenchymal features [327]. Snai1 (Snail), Snai2 (Slug), Twist1, ZEB1, and ZEB2 (also known as Sip-1 and Zfhx1b) are core EMT transcription factors (EMT-TFs) [328]. These core EMT-TFs are mechanically activated by TGFβ–Smads, Wnt/β-Catenin signaling, epidermal growth factor (EGF)/fibroblast growth factor (FGF)–receptor tyrosine kinase (RTK) signaling, Notch signaling, and the MAPK pathway, further initiating EMT-associated changes in gene expression, such as the suppression of E-cadherin and ZO-1 and the activation of N-Cadherin, MMPs, integrins, and fibronectin [329].

**Fig. 6:**
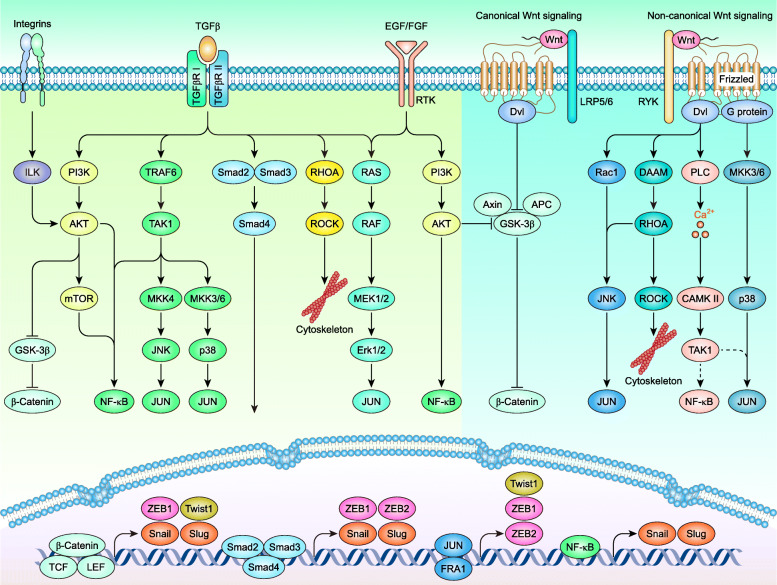
Wnt signaling in the EMT-dependent metastasis of breast cancer. (adapted from [329, 339, 340])

The overexpression of core EMT-TFs has been observed in primary invasive breast cancer and is usually associated with a poor prognosis [327, 330–333]. Specifically, Snail [224], Slug [16, 334], Twist1 [15], ZEB1, and ZEB2 [333] are commonly overexpressed in BLBCs. While Snail [322, 331], Slug [321], Twist1 [335], and ZEB1 [336] are direct targets of Wnt/β-Catenin signaling in breast cancer, ZEB2 is inclined to be an upstream factor of Wnt/β-Catenin signaling [337, 338]. Twist1, ZEB1, and ZEB2 are also induced by the MAPK pathway and may be mediated through TGFβ and noncanonical Wnt signaling [329] (Fig. 6). Overexpressed EMT-TFs suppress the expression of E-cadherin, leading to the release of β-Catenin from the cytomembrane into the cytoplasm (Fig. 1). Free β-Catenin, in turn, promotes the expression of EMT-TFs, thereby forming a positive feedback loop. In addition, Wnt5a/b and Fzd2 drive EMT through a noncanonical Wnt pathway that includes Fyn and Stat3 [22]. The early dissemination and metastasis of Her-2^+^ breast cancer are also driven by the noncanonical Wnt (Wnt5a, Wnt5b, and Wnt11)-dependent EMT-like pathway [11]. Wnt-driven EMT-TF expression further regulates the morphogenesis of breast cancer cells [341] (such as the formation of lamellipodia [342]) and directly secretes MMPs, thereby acquiring migratory and invasive properties. Indeed, EMT also contributes to chemoresistance [343], stem cell properties [344], and immunosuppression [345].

### Wnt signaling in the inter- and intratumoral heterogeneity of breast cancer

Extensive molecular and cellular heterogeneity exists in human breast cancer tissues [312, 346] and determines the diversity of pathological features, prognoses, and responses to available therapy [347]. The heterogeneity of breast cancer involves complicated concepts, termed intertumoral heterogeneity (tumors from different patients), mammary epithelial differentiation hierarchy, intertumoral heterogeneity (within a single tumor), and breast cancer stem cells (BCSCs) [348].

The intertumoral heterogeneity of breast cancer can be explained by a mammary epithelial differentiation hierarchy theory by which different mammary epithelial cell subpopulations that reside in mammary ducts provide a repertoire for intertumoral heterogeneity. Two hypothetical models of mammary epithelial differentiation hierarchy have been established based on gene expression profiling, and the difference lies in whether adult quiescent mammary stem cells (MaSCs) exist [349]. Cumulative evidence indicates that adult bipotent or multipotent quiescent MaSCs, such as LGR5^+^Tspan8^high^ MaSCs [350], may reside within the adult mammary gland [351, 352].

Wnt signaling executes cardinal roles in maintaining the phenotype of MaSCs [349]. Mouse MaSCs are identified by the surface marker Lin^−^CD24^+^CD29^high^ subpopulation, which is expanded in MMTV-Wnt1-induced premalignant mammary tissue [353]. On the other hand, human stem-like cells identified by the surface marker Lin^−^CD10^−^CD24^−^ProCr^+^CD44^+^ subpopulation have been identified in both normal human mammary epithelium and breast carcinomas [21]. Notably, both protein C receptor (ProCr) and CD44 are targets of Wnt/β-catenin signaling [21, 354]. LRG5 is not only a coreceptor (Fig. 1) but also a target of Wnt/β-catenin signaling [355]. LRG5^+^ mammary epithelial cells contribute to the reconstitution of an entire mammary gland, suggesting that LRG5 is a potent biomarker of MaSCs [356]. MMP3, as an extracellular regulator of the Wnt signaling pathway, is necessary for the phenotype and activity maintenance of MaSCs [41]. Axin2 is not only a target but also a negative feedback regulator of Wnt signaling (Fig. 1) and is therefore sensitized to Wnt signals. The Wnt-responsive cell population with Axin2^+^ is enriched for MaSCs in the adult mammary gland [357, 358]. In addition, the macrophages that receive the Notch pathway ligand Dll1 from MaSCs, together with Gli2^+^ stromal cells, govern MaSCs by secreting Wnts and other paracrine factors [359, 360]. These data demonstrate that Wnt signaling is essential for MaSCs to maintain their phenotype and self-renewal.

**Fig. 7:**
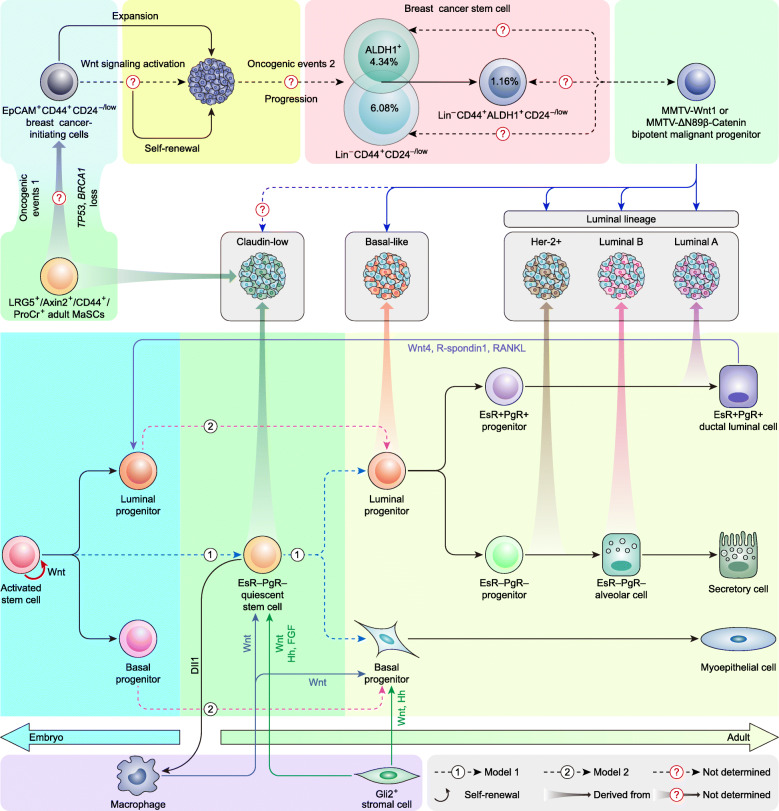
Wnt signaling in the inter- and intratumoral heterogeneity of breast cancer. (adapted from [349])

A comparison of the gene signatures between normal mammary epithelial subpopulations and breast cancer subtypes implied that the claudin-low cancer subtype is remarkably similar to LGR5^+^Tspan8^high^ MaSCs [349, 350]. In contrast, the basal-like cancer subtype shares great similarity to the luminal progenitor subpopulation [361, 362]. The Her-2^+^, luminal B, and luminal A cancer subtypes reflect different cell subtypes within the luminal lineage and in turn gradually lose differentiation capacity. Therefore, the luminal A subtype is closest to mature luminal cells, while the Her-2^+^ and luminal B subtypes likely originate in cells restricted to the luminal lineage [349]. These findings suggest a hypothesis that various breast cancer subtypes (intertumoral heterogeneity) are derived from different mammary epithelial cell subpopulations [363] (Fig. 7). Although Wnt signaling controls various aspects of mammary gland development and differentiation during both embryogenesis and postnatal life [358], this hypothesis is derived from the conjecture of comparative genomics rather than facts. More sophisticated lineage tracing systems may be required to address this question in the future.

Two models have been proposed to account for the intratumoral heterogeneity of breast cancer. The clonal evolution model explains intratumor heterogeneity as a result of natural selection and uses stochastic mutations as a platform. Advantageous clones differ in time and space within an individual tumor and thereby contribute to the intratumoral heterogeneity of breast cancer [348]. The cancer stem cell model hypothesizes that the intratumoral heterogeneity is derived from common malignant self-renewing cells that can generate the full repertoire of tumor cells (i.e., BCSCs). BCSCs are hypothesized to be breast cancer-initiating cells (BCICs) that undergo a second oncogenic event by which BCSCs gain the ability of sustained propagation, whereas BCICs are hypothesized to be MaSCs that undergo one oncogenic event [364]. BCSCs were initially identified by surface markers as Lin^−^CD44^+^CD24^−/low^ [365]. Subsequently, an aldehyde dehydrogenase 1 (ALDH1)^+^ BCSC population capable of self-renewal and of generating tumors that recapitulate the heterogeneity of the parental tumor was identified [366]. Of note, a portion of Lin^−^CD44^+^CD24^−/low^ BCSCs overlap with ALDH1^+^ BCSCs, and Lin^−^CD44^+^CD24^−/low^ALDH1^+^ BCSCs display a more tumorigenic feature [366] (Fig. 7).

Wnt signaling is critical not only to the phenotypic maintenance of BCSCs but also to MaSC–BCSC transformation. CD44 is a well-known target of Wnt/β-catenin signaling and contributes the ‘stemness’ properties to BCSCs [21]. The depletion of CD44 effectively prevents aggregation, blocks lung metastasis, and impairs the ‘stemness’ of circulating breast tumor cells [367]. ProCr is another target of Wnt/β-catenin signaling, and ProCr^+^ MaSCs represent one of the origins of BCSCs [354]. Intriguingly, 100% of CD44^+^ breast tumor cells are positive for ProCr [21]. Indeed, the expression level of CD44 is also controlled by noncanonical Wnt5a [15] and Wnt5b [16]. As discussed above, ALDH1^+^ BCSCs represent another important subpopulation of BCSCs [366]. ALDH1 is not a direct target of Wnt/β-catenin signaling; however, its activity is controlled by syndecan-1, a coreceptor of noncanonical Wnt–PCP signaling (Fig. 2 c), and the BCSC phenotype that is characterized by ALDH1 activity and CD44^+^CD24^−/low^ is reduced upon Syndecan-1 knockdown [248]. These findings suggest that the phenotypic maintenance of BCSCs is governed jointly by both canonical and noncanonical Wnt signaling.

The constitutive overexpression of Wnt1 in the mammary gland directly gives rise to tumors [5], suggesting that Wnt signaling activation is an independent oncogenic event. MMTV-Wnt1- and MMTV-ΔN89β-Catenin-induced tumors contain differentiated cells of both luminal and basal lineages, suggesting that the precursor of Wnt1- and ΔN89β-Catenin-induced tumors is a bipotential stem cell [24, 368]. The loss of *Pten* accelerates the dysregulation of this subpopulation during tumor initiation [288]. Intriguingly, MMP3, as a regulator of Wnt signaling, maintains the phenotype of MaSCs on one side [41] and promotes mammary carcinogenesis on the other [369]. Additionally, the Lin^−^CD24^+^CD29^high^ MaSC subpopulation is expanded in MMTV-Wnt1-induced premalignant mammary tissue [353]. These findings indicate that Wnt signaling is the principal driver in MaSC–BCSC transformation. However, the relationship between Wnt1/ΔN89β-Catenin-induced bipotential stem cells and Lin^−^CD44^+^CD24^−/low^ or ALDH1^+^ BCSCs remains obscure (Fig. 7).

### Wnt signaling in breast cancer drug resistance

**Fig. 8:**
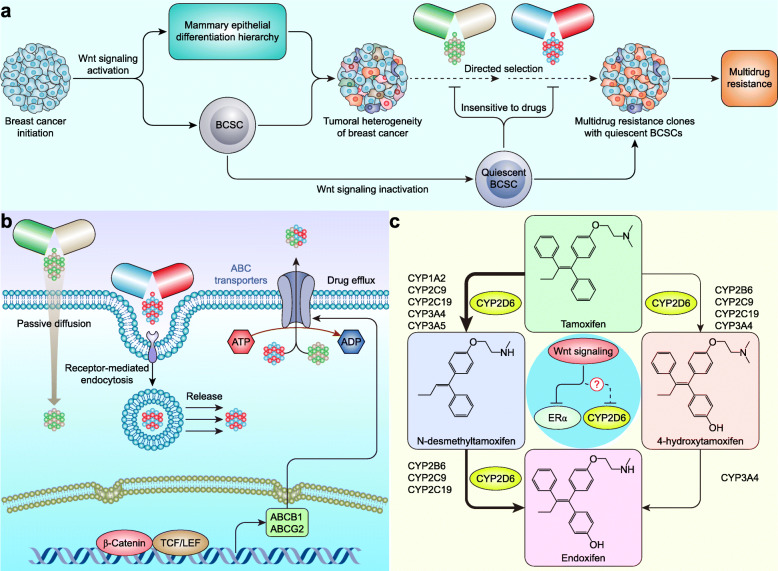
Wnt signaling in breast cancer drug resistance. **a** Wnt signaling-induced tumoral heterogeneity involves the drug resistance of breast cancer. **b** The APC transporters P-gp (encoded by *ABCB1*) and BCRP (encoded by *ABCG2*), which are involved in drug efflux, are targets of Wnt signaling in breast cancer. **c** Wnt signaling involves endocrine resistance in breast cancer. The thickness of the arrow represents the relative contribution of each pathway to the overall oxidation of tamoxifen (adapted from [374])

Drug resistance in cancer is considered to be a multifaceted problem involving tumoral heterogeneity, drug efflux/inactivation, survival pathway activation, etc. [370]. The Goldie-Coldman hypothesis explains drug resistance as a result of directed selection and uses heterogeneous tumor cell clones with various mutations as a platform [371, 372]. Drug-resistant clones survive and expand under toxic drug stress; on the other hand, Wnt signaling inactivation causes BCSCs to enter a quiescent state that is insensitive to drugs [352, 373], thereby leading to multidrug resistance. The Wnt signaling-mediated mammary epithelial differentiation hierarchy and the formation and self-renewal of BCSCs are drivers of the tumoral heterogeneity of breast cancer (Fig. 8 a).

Drug efflux from cancer cells mediated by ATP-binding cassette (ABC) transporters is another vital pathway in the drug resistance of breast cancer. P-glycoprotein (P-gp, also known as MDR1, encoded by *ABCB1*), multidrug resistance protein 1–5 (MRP1–5, encoded by *ABCC1–5*), and breast cancer resistance protein (BCRP, encoded by *ABCG2*) are well-defined ABC transporters that are involved in the transport of clinically relevant drugs [375]. PYGO2 is a coactivator in Wnt/β-catenin signaling (Fig. 1) that mediates chemoresistance by activating MDR1 expression in breast cancer [376]. Caveolin 1 is overexpressed and amplified in a subset of basal-like and metaplastic breast carcinomas [377, 378] and promotes drug resistance by increasing ABCG2 expression in a Wnt/β-catenin signaling-dependent manner [379, 380] (Fig. 8 b).

EsR-positive breast cancers account for nearly 80% of all breast cancer cases [1], and approximately 50% of mortalities arise from EsR-positive breast tumors [381]. EsR is a ligand-inducible transcription factor that contains a central DNA binding domain, an intrinsically disordered N-terminal activation function 1 (AF1) domain, and a C-terminal ligand-binding domain (LBD) [381]. Tamoxifen was the first clinically approved EsR-targeted drug and competes with 17β-estradiol (E2) for EsR binding and prevents LBD-mediated coactivator recruitment, thereby impairing the transcriptional activity of EsR [381]. The primary (4-hydroxytamoxifen) and secondary (endoxifen) metabolites of tamoxifen mediated by the cytochrome P450 system are more potent than tamoxifen itself [374]. Cytochrome P450 2D6 (CYP2D6) is undoubtedly the key enzyme for endoxifen generation. One-third of women treated with tamoxifen for 5 years experience recurrence within 15 years, and endocrine-resistant disease may account for 25% of all breast cancers [382]. Inactive CYP2D6 that fails to convert tamoxifen to endoxifen and the lack of ERα expression are primary mechanisms of endocrine resistance [383]. Intriguingly, both canonical and noncanonical Wnt signaling pathways are activated in tamoxifen-resistant breast cancer cells, and Wnt3a increases the resistance of EsR^+^ breast cancer cells to tamoxifen treatment [384]. Furthermore, Sox2 is increased in tamoxifen-resistant breast cancer cells and negatively correlated with ERα expression. Sox2 also maintains the phenotype of breast cancer stem/progenitor cells by activating Wnt signaling, thereby rendering EsR^+^ breast cancer cells insensitive to tamoxifen treatment [14]. Although there is no direct evidence to prove the relationship between Wnt signaling and CYP2D6 activity or tamoxifen metabolism, some studies indicate that such a relationship may exist. Endoxifen levels are 20% lower during winter months than return to mean levels across seasons and are associated with low vitamin D3 levels; thus, vitamin D3 may maintain endoxifen levels by increasing CYP2D6 activity [385]. Indeed, vitamin D3 can regulate intestinal CYP3A4 expression through the binding of the vitamin D receptor (VDR)-retinoid X receptor (RXR) heterodimer to the ER6 motif of the *CYP3A4* promoter [386, 387]. On the other hand, vitamin D3 increases tamoxifen sensitivity by inhibiting Wnt/β-catenin signaling [388] (Fig. 8 c). Of note, vitamin D3 has been proven to be a potent disruptor of β-Catenin/TCF [389, 390]. Endoxifen is also a substrate of the efflux transporter MDR1 [391], a target of Wnt signaling, as we discussed above [376]. These findings indicate that Wnt signaling is involved in endocrine therapy resistance in breast cancer.

Immunotherapy for breast cancer has attracted wide attention and interest, and immune checkpoint blockade is the most investigated form in more than 290 ongoing clinical trials of breast cancer immunotherapy [392]. Undoubtedly, PD-1/PD-L1 and CTLA-4 are the most attractive targets among immune checkpoint inhibitors [393]. Indeed, more than 40% of TNBCs are PD-L1 positive, and the anti-PD-L1 monoclonal antibody atezolizumab has been approved by the FDA for advanced or metastatic PD-L1-positive TNBC [394]. Nevertheless, approximately 40% of PD-L1-positive TNBCs exhibit a poor response to atezolizumab plus nab-paclitaxel treatment. Given the 10.3% complete response rate in PD-L1-positive TNBC, much more effort may be needed [394]. Wnt signaling not only controls the expression of PD-L1 [306] and CTLA-4 [307] but also blocks the tumor-immune cycle at all steps [395]. β-Catenin/STT3-dependent PD-L1 N-glycosylation stabilizes and upregulates PD-L1, which promotes breast cancer immune evasion [396]. Moreover, MMTV-Wnt1 breast tumors are classified as ‘cold tumors’, suggesting that Wnt signaling mediates immunotherapy resistance [397]. Thus, targeting Wnt signaling is a potential strategy to enhance the efficacy of cancer immunotherapy (Fig. 5 d).

### Molecular agents targeting the Wnt signaling pathway in breast cancer

Hundreds of inhibitors have been developed over the past few decades. These inhibitors are generally focused on targeting Porcupine, Fzds, DVLs, TNKS 1/2, and β-Catenin/TCF or β-Catenin/coactivators.

**Fig. 9:**
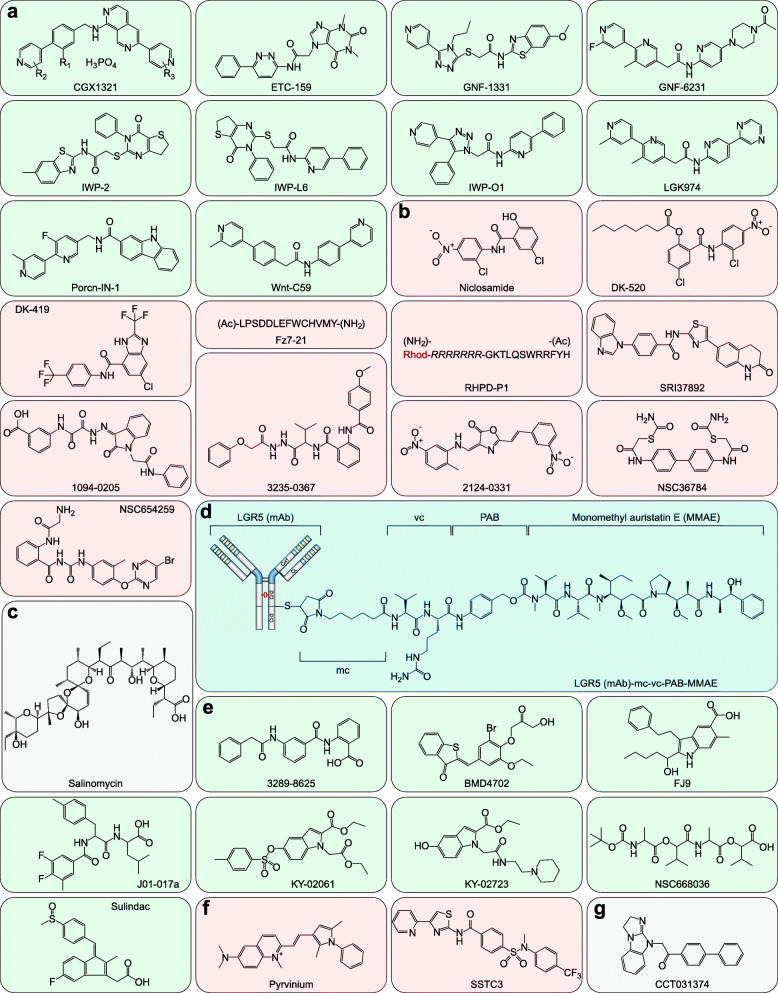
Selected Wnt signaling inhibitors (Part 1). **a** Porcupine inhibitors. **b** Fzd inhibitors. **c** Wnt/Fzd/LRP inhibitor. **d** LGR5-specific antibody-drug conjugate. **e** Dvl inhibitors. **f** CK1α agonists. **g** GSK-3β agonist

Porcupine inhibitors have recently received great attention because of their broad-spectrum Wnt-targeted and anticancer activity. LGK974, a representative Porcupine inhibitor, is being tested in a phase I clinical trial in patients with TNBC and other Wnt-driven cancers [16, 63, 398]. Of note, GNF-6231, Porcn-IN-1, and Wnt-C59 are LGK974 analogs (Fig. 9 a and Table 4). Perturbation of Wnt–Fzd interactions is another strategy to block Wnt signaling transduction. OMP-54F28 is an Fc fusion protein that contains the extracellular N-terminal cysteine-rich domain (CRD) of Fzd8 and serves as a decoy receptor, competing with Fzd8 for all Wnts [17, 408, 409]. R-spondins are indirect activators of Wnt signaling; OMP-131R10, an anti-R-spondin-3 antibody, has entered a phase I clinical trial for solid tumor treatment (Table 5).

**Table 4:** Inhibitors of Porcupine

Compound	IC_50_	Development stage	Ref.
CGX1321	1.0 nM	Phase I (NCT03507998): Gastrointestinal tumors; Phase I (NCT02675946): Solid tumors	[399, 400]
ETC-159	2.9 nM	Phase I (NCT02521844): Advanced solid tumors	[401]
LGK974	0.1 nM	Phase I (NCT01351103): TNBC and other cancers	[16, 63, 398]
GNF-1331	12 nM	Preclinical	[402]
GNF-6231	0.8 nM	Preclinical	[402]
IWP2	27 nM	Preclinical	[384, 403, 404]
IWP-L6	0.5 nM	Preclinical	[405]
IWP-O1	80 pM	Preclinical	[406]
Porcn-IN-1	0.5 ± 0.2 nM	–	[407]
Wnt-C59	74 pM	Preclinical	[398]

**Table 5:** Inhibitors of Fzds and related factors

Compound	Target	IC_50_	Development stage	Ref.
OMP-54F28 (Fzd8-Fc fusion)	Wnts	ND	Phase I (NCT02069145): Hepatocellular cancer; Phase I (NCT02092363): Ovarian cancer; Phase I (NCT02050178): Pancreatic cancer; Phase I (NCT01608867): Solid tumors	[408]
OMP-131R10 (mAb)	R-spondin3	ND	Phase I (NCT02482441): Solid tumors	[410]
Niclosamide	Fzd1	0.5 ± 0.05 μM	FDA-approved antihelminth; Phase I (NCT03123978): Prostate cancer; Phase II (NCT02519582): Colorectal cancer; Phase II (NCT02807805): Prostate cancer	[411]
DK-520	Fzd1	0.23 ± 0.06 μM	Preclinical	[412]
DK-419	Fzd1	0.19 ± 0.08 μM	Preclinical	[413]
OMP-18R5 (mAb)	Fzd1/2/5/7/8	ND	Phase I (NCT01345201): Solid tumors; Phase I (NCT02005315): Pancreatic cancer; Phase I (NCT01957007): NSCLC; Phase I (NCT01973309): Breast cancer	[414]
IgG-2919 (mAb)	Fzd5/8	ND	Preclinical	[415]
Fz7–21	Fzd7	50–100 nM	Preclinical	[416]
RHPD-P1		7–40 μM	Preclinical	[417]
SRI37892		0.66 μM	Preclinical	[418]
1094–0205	Fzd8	5.0 ± 1.1 μM	Preclinical	[419]
2124–0331		10.4 ± 2.0 μM	Preclinical	[419]
3235–0367		7.1 ± 1.4 μM	Preclinical	[419]
NSC36784		6.5 ± 0.9 μM	Preclinical	[419]
NSC654259		5.7 ± 1.2 μM	Preclinical	[419]
OTSA101-DTPA-^111^In; OTSA101-DTPA-^90^Y	Fzd10	ND	Phase I (NCT04176016): Synovial sarcoma	[420, 421]
Salinomycin	Wnt/Fzd/LRP	163 nM	Preclinical	[422]
LGR5 (mAb)-mc-vc-PAB-MMAE	LGR5	ND	Preclinical	[423]

*ND* not determined

Targeting Fzds is also a mainstream strategy to block Wnt signaling. Niclosamide is an FDA-approved antihelminth. As an inhibitor of Fzd1, it has entered phase I/II clinical trials and could be the most promising Fzd inhibitor [411]. OMP-18R5, an antibody that targets multiple Fzds, binds to 5 of the 10 Fzds and is a potential drug for breast cancer and other solid tumors [414]. In addition, OTSA101-DTPA-^111^In and OTSA101-DTPA-^90^Y are humanized chimeric anti-Fzd10 antibodies (named OTSA-101) that are radiolabeled with Indium 111 and Yttrium 90, respectively. OTSA101-DTPA-^111^In is a promising Fzd10-targeted single-photon emission computed tomography (SPECT) imaging agent, while OTSA101-DTPA-^90^Y is a potent Fzd10-targeted agent for metastatic synovial sarcoma radiotherapy [420, 421]. Salinomycin is an FDA-approved supplement in poultry feed and kills BCSCs selectively [424]. It was subsequently proven to block Wnt-induced LRP phosphorylation [422]. LGR5 (mAb)-mc-vc-PAB-MMAE is an LGR5-specific antibody-drug conjugate (ADC). Monomethyl auristatin E (MMAE) is a tubulin-inhibiting cytotoxic drug that kills LGR5-positive cancer cells selectively [423] (Fig. 9 b-d and Table 5).

Dvls, as the main intracellular effectors of the Wnt/Fzd/LRP complex, are ideal targets. Quite a few small-molecule inhibitors have been developed for Dvl inhibition. Sulindac is the most promising Dvl inhibitor among these small-molecule inhibitors. It is an FDA-approved nonsteroidal anti-inflammatory drug that has been shown to have clinically significant anticancer effects. Sulindac is an inhibitor of not only cyclooxygenase 1/2 (COX1/2) [425] but also Dvl in the PDZ domain [426]. It is hypothesized to suppress tumor growth by blocking Dvl activity rather than prostaglandin synthesis [427, 428] (Fig. 9 e and Table 6).

**Table 6:** Inhibitors of Dvls

Compound	IC_50_	Development stage	Ref.
3289–8625	12.5 μM	Preclinical	[429]
BMD4702	ND	Preclinical	[430]
FJ9	ND	Preclinical	[431]
J01-017a	1.5 ± 0.2 μM	Preclinical	[432]
KY-02061	24 μM	Preclinical	[433]
KY-02327	3.1 μM	Preclinical	[433]
NSC668036	ND	Preclinical	[434]
Sulindac	ND	FDA-approved nonsteroidal anti-inflammatory drug; Phase I (NCT00245024): Breast cancer; Phase II (NCT00039520): Breast cancer; Phase III (NCT01349881): Colorectal neoplasms	[426]

**Fig. 10:**
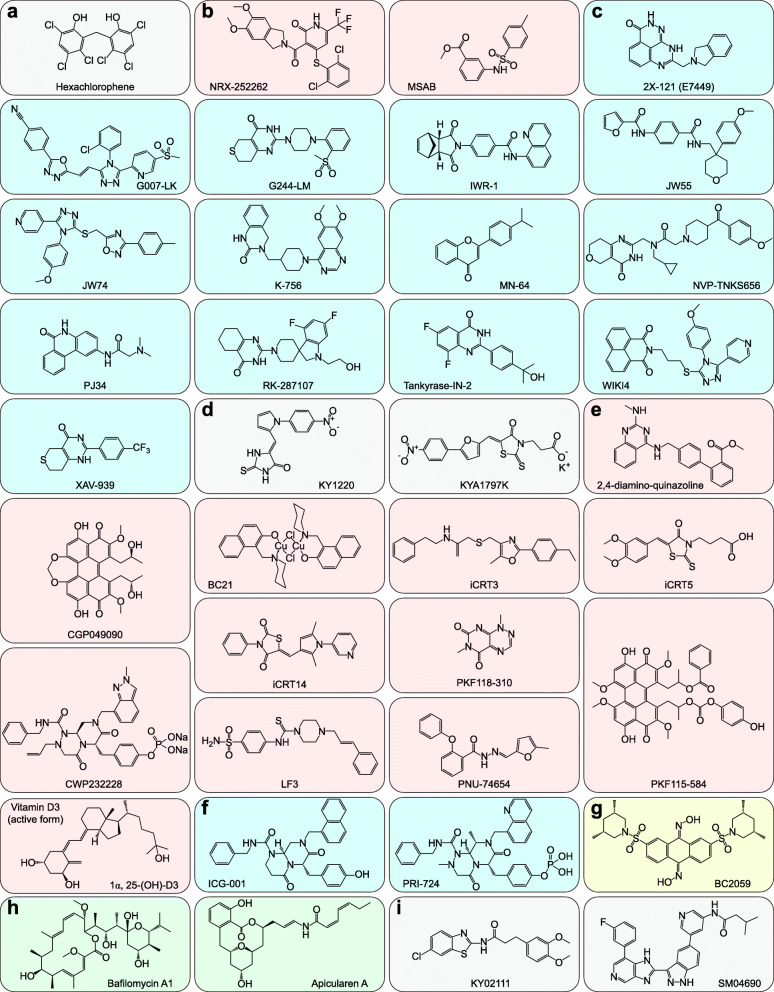
Selected Wnt signaling inhibitors (Part 2). **a** Siah-1 agonist. **b** β-Catenin destabilizers. **c** TNKS1/2 inhibitors. **d** Axin stabilizers. **e** β- Catenin/TCF disruptors. **f** β-Catenin/CBP disruptors. **g** β-Catenin/TBL1 disruptor. **h** V-ATPase inhibitors. **i** Wnt signaling inhibitors with an unknown target

β-Catenin is the key to canonical Wnt signaling. The direct inhibition or degradation of β-Catenin is assuredly an effective strategy. Only two small molecules (MSAB [435] and NRX-252262 [436]) that directly target β-Catenin have been identified to date. Other small molecules that target β-Catenin by enhancing the formation of the destruction complex, such as activating CK1α [437–439], GSK-3β [440], and Axin [441], have also received full attention. Another destruction complex-independent strategy is activating Siah-1-induced β-Catenin degradation with hexachlorophene [442]. Given the critical function of Axin degradation mediated by TNKS1/2, various small-molecule inhibitors have been developed to inhibit TNKS1/2. 2X-121 (also known as E7449), the most promising TNKS1/2 inhibitor, has entered phase I/II clinical trials for breast cancer and ovarian cancer treatment [443, 444] (Fig. 9 f-g, Fig. 10 a-c and Table 7).

**Table 7:** Small molecules that degrade β-Catenin at the cytoplasmic level

Compound	Target	IC_50_/EC_50_	Development stage	Ref.
Pyrvinium	CK1α	10 nM	FDA-approved antihelminth	[437]
SSTC3		30 nM	Preclinical	[438]
CCT031374	GSK-3β	6.1 μM	Preclinical	[440]
Hexachlorophene	Siah-1	7.03 μM	Preclinical	[442]
MSAB	β-Catenin	0.583 μM	Preclinical	[435]
NRX-252262		3.8 ± 0.2 nM	Preclinical	[436]
2X-121 (E7449)	TNKS1/2	50 nM	Phase II (NCT03562832): Breast cancer; Phase II (NCT03878849): Ovarian cancer; Phase I/II (NCT01618136): TNBC and other cancers;	[443, 444]
G007-LK		0.08 μM	Preclinical	[445, 446]
G244-LM		0.11 μM	Preclinical	[445]
IWR-1		0.18 μM	Preclinical	[403, 447]
JW55		470 nM	Preclinical	[448]
JW74		420 nM	Preclinical	[449]
K-756		31 nM (TNKS1), 36 nM (TNKS2)	Preclinical	[450]
MN-64		6 nM (TNKS1), 72 nM (TNKS2)	Preclinical	[451]
NVP-TNKS656		6 nM (TNKS2)	Preclinical	[452]
PJ34		1 μM (TNKS1)	Preclinical	[453]
RK-287107		14.3 nM (TNKS1), 10.6 nM (TNKS2)	Preclinical	[454]
Tankyrase-IN-2		10 nM (TNKS1), 7 nM (TNKS2)	Preclinical	[455]
WIKI4		26 nM (TNKS2)	Preclinical	[456, 457]
XAV939		5 nM (TNKS1), 2 nM (TNKS2)	Preclinical	[133, 451, 458, 459]
KY1220	Axin	2.1 μM	Preclinical	[441]
KYA1797K		0.75 μM	Preclinical	[441]

Nuclear β-Catenin serves as a scaffold for its coactivators to bind rather than as an independent transcription factor. Thus, disrupting the interaction between β-Catenin and its coactivators is also a potent strategy to block Wnt signaling transduction. β-Catenin/TCF becomes the primary target for disruption. However, vitamin D3, as a potent β-Catenin/TCF disruptor [389, 390], has been proven to be invalid for cancer prevention and treatment [460, 461]. Disrupting the interaction between β-Catenin and BCL9 or CBP is an optional strategy to block the transcription of Wnt target genes. PRI-724, as a β-Catenin/CBP disruptor, has entered a phase I clinical trial for advanced solid tumor treatment [462]. Transducin β-like protein 1 (TBL1)–TBL1-related protein (TBLR1) and β-Catenin recruit each other, displacing the corepressors TLE and HDAC1 and resulting in the stimulation of Wnt target gene transcription [463]. BC2059 is a β-Catenin/TBL1 disruptor and has entered a phase I clinical trial for desmoid tumor treatment [464]. Intriguingly, apicularen A and bafilomycin A1, as vacuolar H^+^-adenosine triphosphatase (V-ATPase) inhibitors, effectively inhibit Wnt signaling [465]. In addition, although the direct targets of KY02111 [466] and SM04690 remain unknown, SM04690 has entered a phase II clinical trial for knee osteoarthritis treatment [467] (Fig. 10 d-i and Table 8).

**Table 8:** β-Catenin inhibitors in cancers

Compound	Target	IC_50_/EC_50_	Development stage	Ref.
2,4-diamino-quinazoline	β-Catenin/ TCF	0.6 μM	Preclinical	[468]
BC21		15 μM	Preclinical	[469]
CGP049090		8.7 μM	Preclinical	[470, 471]
CWP232228		0.8 ~ 2 μM	Preclinical	[472]
iCRT3		8.2 nM	Preclinical	[473]
iCRT5		18.7 nM	Preclinical	[473]
iCRT14		40.3 nM	Preclinical	[473]
LF3		1.65 μM	Preclinical	[474]
PKF115–584		3.2 μM	Preclinical	[470, 471]
PKF118–310		0.8 μM	Preclinical	[470, 471]
PNU-74654		ND	Preclinical	[475]
Vitamin D3		ND	Phase II (NCT01948128): Breast cancer; Phase III (NCT01169259): Cancer and cardiovascular disease; Phase III (NCT02786875): Breast cancer	[389, 390]
SAH-BCL9	β-Catenin/ BCL9	ND	Preclinical	[476]
ICG-001	β-Catenin/ CBP	3.0 μM	Preclinical	[477]
PRI-724		ND	Phase I (NCT01302405): Advanced solid tumors	[462]
BC2059	β-Catenin/ TBL1	ND	Phase I (NCT03459469): Desmoid tumor	[464]
Apicularen A	V-ATPase	20 nM	Preclinical	[465, 478]
Bafilomycin A1		0.44 nM	Preclinical	[465]
KY02111	Unknown	ND	Preclinical	[466]
SM04690		19.5 nM	Phase II (NCT03706521): Knee osteoarthritis	[467]

### Challenges and opportunities

Wnt signaling activation in colorectal cancer is induced mainly by *APC* (73%) and *CTNNB1* (5%) mutations [217], suggesting that canonical Wnt/β-Catenin signaling is the leading form of Wnt signaling in colorectal cancer. By contrast, Wnt signaling activation in breast cancer is more complicated and often involves dual effects of canonical and noncanonical Wnt signaling [22, 189, 479]. Although extensive research has been carried out, it is still unclear whether Wnt signaling can be druggable successfully for the therapeutic purposes of breast cancer.

The safety and effectiveness of Wnt signaling-targeted drugs is the most concerning issue that we have to face. Abrogation of the aberrant ‘dark side’ of Wnt signaling in breast cancer without interfering with its crucial role in tissue homeostasis and repair is undoubtedly the most desirable clinical outcome [17]. However, the majority of available Wnt inhibitors (such as LGK974) are broad spectrum, and it is challenging to achieve balance by controlling the dosage or appropriate time of drug administration. In addition, the effectiveness of Wnt signaling-targeted drugs needs to be further confirmed in clinical trials. Of note, very few inhibitors of noncanonical Wnt signaling have been identified or developed. Extensive crosstalk between noncanonical Wnt signaling and many other signaling pathways exists, making it difficult to specifically target Wnt signaling. ROCK, as a critical component of Wnt–PCP signaling (Fig. 2c), can be inhibited by the small molecule fasudil, thereby blocking Wnt–PCP signaling [480].

**Fig. 11:**
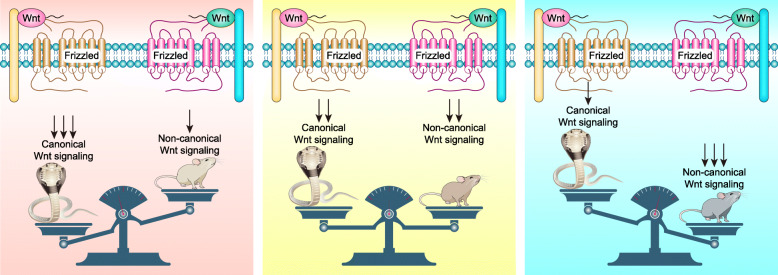
The bipolar seesaw model between canonical and noncanonical Wnt signaling. (some compositional elements of this figure were obtained from https://www.16pic.com and reference [486])

Furthermore, the mechanism of balance between canonical and noncanonical Wnt signaling should be addressed. For example, Wnt5a antagonizes canonical Wnt/β-Catenin signaling and exhibits tumor-suppressive activity in some circumstances [221, 481–483], but other studies have reported that Wnt5a controls both canonical and noncanonical Wnt signaling [15, 16, 484]. Nusse et al. explained that Wnt5a activates or inhibits β-Catenin–TCF signaling depending on the receptor context [485]. However, the switch and balance between canonical and noncanonical Wnt signaling may involve more profound mechanisms. We propose a bipolar seesaw model to illustrate this ebb and flow: various Wnt ligands and their receptors form a unique combination, and the activation of canonical or noncanonical Wnt signaling depends on this unique combination. Fzds–Dvls complex-guided downstream kinase cascades differ in canonical and noncanonical Wnt signaling. The switch mechanism may exist not only for the Wnts–Fzds complex but also for downstream kinase cascades (Fig. 11).

Despite the potential safety and effectiveness concerns regarding the therapeutic targeting of Wnt signaling in breast cancer, constantly emerging novel inhibitors and ongoing clinical trials may ameliorate these issues. Additionally, the application of small-molecule libraries such as Pfizer compounds and molecular docking algorithms based on structural information may accelerate this process. The decryption of underlying mechanisms, including the molecular subtype, tumor stage, and microenvironment context-dependent Wnt signaling activation, as well as the switch and balance between canonical or noncanonical Wnt signaling, is undoubtedly the rationale to ameliorate the safety and effectiveness of Wnt-targeted therapy, especially for breast cancer and other Wnt-driven cancers.

## Conclusions

Accumulating evidence corroborates that the aberrant activation of Wnt signaling exists from breast tumor initiation to distant metastasis. An increasing number of Wnt-targeted small molecules and biologics have entered clinical trials for breast cancer treatment, suggesting that Wnt signaling is an attractive target. The identification of accurate targets and the development of safe and effective drugs are rationales for subsequent clinical trials to determine the appropriate dosage and time of drug administration. Regarding the evolution of Wnt inhibitors, monoclonal antibodies and ADCs will be the mainstream drugs in the future, which is in line with the trend of precision medicine and personalized treatment. Given the unique roles of the noncanonical Wnt pathway in breast cancer, more specific inhibitors should be developed in the future.

Although numerous studies have verified that both canonical and noncanonical Wnt signaling pathways are involved in the progression of breast cancer, there are still no available Wnt-targeted inhibitors for breast cancer treatment in a variety of clinical contexts. Efforts to seek suitable means to regulate Wnt signaling in breast cancer and other Wnt-driven cancers are still ongoing, but emerging discoveries suggest that Wnt-targeted therapy will translate soon into real therapies [90].

## Data Availability

Not applicable.

## Abbreviations

7-TM: 7-Transmembrane
ABC transporters: ATP-binding cassette transporters
ADC: Antibody-drug conjugate
AF1: Activation function 1
ALDH1: Aldehyde dehydrogenase 1
AP1: Activator protein 1
APC: Adenomatous polyposis coli
APCs: Antigen-presenting cells
ATF2: Activating transcription factor 2
BCICs: Breast cancer-initiating cells
BCL9: B cell lymphoma 9
BCRP: Breast cancer resistance protein
BCSCs: Breast cancer stem cells
BLBC: Basal-like breast cancer
BL-1: Basal-like 1
BL-2: Basal-like 2
CAFs: Cancer-associated fibroblasts
CAMK II: Calcium calmodulin mediated kinase II
CapZIP: CapZ-interacting protein
CBP: CREB-binding protein
CBY: Chibby
Cdc42: Cell-division cycle 42
Celsr1: Cadherin EGF LAG seven-pass G-type receptor 1
cGMP: Cyclic guanosine monophosphate
CK1α: Casein kinase 1α
COX1/2: Cyclooxygenase 1/2
CRD: Cysteine-rich domain
CTBP: C-terminal binding protein
CTLA-4: Cytotoxic T lymphocyte antigen 4
CYP2D6: Cytochrome P450 2D6
DAAM: Dishevelled-associated activator of morphogenesis
DAG: Diacylglycerol
DCIS: Ductal carcinoma in situ
Dkks: Dickkopf proteins
DIA1: Diaphanous 1
Dvl: Dishevelled
ECM: Extracellular matrix
EGF: Epidermal growth factor
EMT: Epithelial-mesenchymal transition
EMT-TFs: EMT transcription factors
ER: Endoplasmic reticulum
EsR: Estrogen receptor
FasL: Fas ligand
FDA: Food and Drug Administration
FGF: Fibroblast growth factor
FoxP3: Forkhead box protein P3
Fzds: Frizzleds
G-CSF: Granulocyte colony-stimulating factor
GRG: Groucho
GSK-3β: Glycogen synthase kinase 3β
HDACs: Histone deacetylases
Her-2: Human epidermal growth factor receptor 2
LBD: Ligand-binding domain
ICAT: Inhibitor of β-Catenin and TCF
IDC-NST: Invasive ductal carcinoma no-special-type
IDO: Indoleamine 2,3-dioxygenase
IFN-γ: Interferon γ
IGFBP4: Insulin-like growth factor-binding protein 4
IL: Interleukin
ILC: Invasive lobular carcinoma
IM: Immunomodulatory
iNOS: Inducible nitric oxide synthase
InsP_3_: Inositol-1, 4, 5-trisphosphate
InsP_3_Rs: Inositol-1,4,5-trisphosphate receptors
Intu: Inturned
Invs: Inversin
JNK: Jun N-terminal kinase
LAR: Luminal androgen receptor
LCIS: Lobular carcinoma in situ
LEF1: Lymphoid Enhancer Factor 1
LGR5: Leucine-rich repeat-containing G protein-coupled receptor 5
LRPs: Low-density lipoprotein receptor-related proteins
M: Mesenchymal
MaSCs: Mammary stem cells
MHC I: Major histocompatibility complex class I
MKK3/6: Mitogen-activated protein kinase 3/6
MMAE: Monomethyl auristatin E
MMPs: Matrix metalloproteinases
MMTV: Mouse mammary tumor virus
MRLC: Mitogen-activated protein kinase
MRP1–5: Multidrug resistance protein 1–5
MSL: Mesenchymal stem-like
MUSK: Muscle skeletal receptor Tyr kinase
NFAT: Nuclear factor of activated T cells
NLK: Nemo-like kinase
PCP: Planar cell polarity
PDE6: Phosphodiesterase 6
PD-L1: Programmed death-ligand 1
PKG: Protein kinase G
P-gp: P-glycoprotein
PgR: Progesterone receptor
PKC: Protein kinase C
PLC: Phospholipase C
PP2A: Protein phosphatase 2A
ProCr: Protein C receptor
PtdInsP_2_: Phosphatidylinositol-4,5-bisphosphate
PTK7: Protein Tyr kinase 7
PYGO: Pygopus
RANK: Receptor activator of nuclear factor-κB
RANKL: Receptor activator of nuclear factor-κB ligand
RHOA: RAS homologue gene-family member A
ROCK: RHO-associated coiled-coil-containing protein kinase
ROR1/2: Receptor Tyr kinase-like orphan receptor 1/2
RTKs: Receptor tyrosine kinases
RXR: Retinoid X receptor
RYK: Receptor-like tyrosine kinase
SCF^β-TrCP^: Skp1, Cullin1 and F-box protein β-TrCP
SERCAs: Sarcoplasmic/ER Ca^2+^ ATPases
sFRPs: Secreted Frizzled-related proteins
SOCE: Store-operated Ca^2+^ entry
SPECT: Single-photon emission computed tomography
STIM1/2: Stromal interaction molecule 1/2
TAK1: TGFβ-activated protein kinase 1
TAMs: Tumor-associated macrophages
TBL1: Transducin β-like protein 1
TBLR1: TBL1-related protein
TCF: T cell factor
TLE: Transducin-like enhancer
TNBC: Triple-negative breast cancer
TNKS1/2: Tankyrase 1/2
TNF-α: Tumor necrosis factor α
TRAIL: TNF-related apoptosis-inducing ligand
V-ATPase: Vacuolar H^+^-adenosine triphosphatase
Vangl2: Vang-like 2
VDR: Vitamin D receptor
VEGFs: Vascular endothelial growth factors
WIF-1: Wnt inhibitory factor 1
Wnts: Wnt proteins
WTX: Wilms tumor gene on X chromosome
